# 1791. Use of pathogen genomics to monitor global emergence of extensively drug-resistant Salmonella Serotype Typhi

**DOI:** 10.1093/ofid/ofad500.1620

**Published:** 2023-11-27

**Authors:** Nkuchia M Mikanatha, Yezhi Fu, Xin Yin, Christopher E Carr

**Affiliations:** Pennsylvania Department of Health, Harrisburg, Pennsylvania; Penn State College of Agricultural Sciences, University Park, Pennsylvania; Penn State College of Medicine, Hershey, Pennsylvania; Georgia Tech, Atlanta, Georgia

## Abstract

**Background:**

Whole-genome sequencing (WGS) has transformed surveillance and outbreak detection. However, its use to complement resource-intensive epidemiologic investigations of emerging pathogens is limited. Here using WGS data of *S.* Typhi, we investigate the global spread of an emerging pathogen.

**Methods:**

We used NCBI pathogen detection to search for SNP clusters of ceftriaxone-resistant *S*. Typhi (CRT) reported in the US and the National Antimicrobial Monitoring System (NARMSNOW) to analyze antibiotic-resistant trends of *S.* Typhi.

**Results:**

Pathogen Detection Outbreaks of CRT infections were first reported in the US in 2018. We found that the US CRT isolates formed two distinct clusters linked to travel destinations, having close genetic relatedness with those found in Pakistan or Iraq. Specifically, three US CRT isolates (linked to travel to Iraq) only differed from 10 Iraq isolates (2021-2022) by an average of 3 SNPs (**Figure**). Further, two US CRT isolates (linked to travel to Pakistan) only differed from one Pakistan isolate (2022) by an average of 5 SNPs.

NARMS Now

Using 2012-2022 NARMS Now data, we analyzed trends in decreased susceptibility to ciprofloxacin (DSC) and resistance to ≥3 antibiotics recommended for treatment (MDR) among *S.* Typhi. We also compared the resistance trend pre-pandemic and after 2020. Our analysis included a total of 3,633 *S.* Typhi isolates. The percentage of DSC isolates increased from 68.5% (2012) to 85.1% (2022), while the percentage of MDR isolates increased from 8.9% to 20.1%. The 3-year (2017-2019) pre-pandemic DSC average was 77.5% and 80.5% after (2020-2022). For MDR isolates, it was 12.4% and 21.8%.

Close genetic relatedness between three US outbreak associated ceftriaxone-resistant Typhi strains (linked to travel to Iraq; highlighted in red) and 10 strains isolated in Iraq (highlighted in red).

**Figure:**
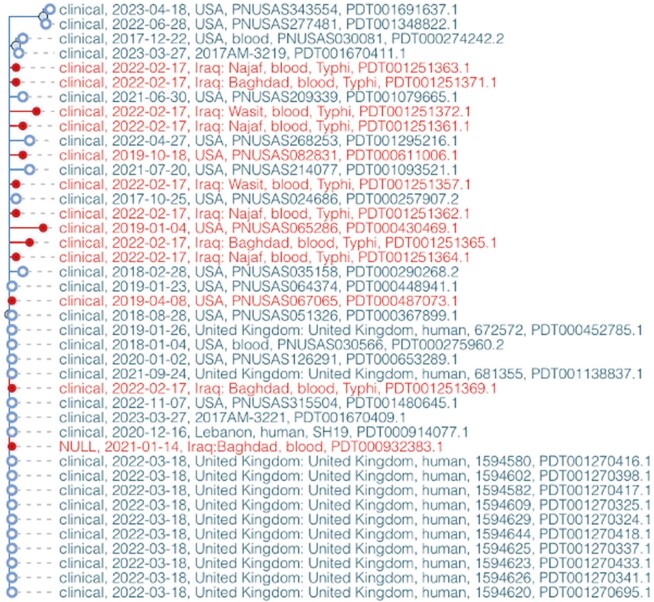

Note the strains are collected in different years. The single nucleotide polymorphism (SNP) tree is automatically generated in the NCBI Pathogen Detection database when sequencing data are submitted to the database. The average SNP distance for the red highlighted isolates in the tree is three (SNP distance range 0∼9).

**Conclusion:**

The SNP clusters indicate the likely international spread of resistant *S.* Typhi strains in travelers. Resistance to ciprofloxacin is increasing as is resistance to multiple antimicrobials. Travel restrictions imposed during the pandemic did not appreciatively alter resistance trends. These findings indicate the need for global cooperation in addressing antimicrobial resistance and emerging pathogens.

**Disclosures:**

**All Authors**: No reported disclosures

